# Natural Occurrence of *Alternaria* Fungi and Associated Mycotoxins in Small-Grain Cereals from The Urals and West Siberia Regions of Russia

**DOI:** 10.3390/toxins13100681

**Published:** 2021-09-25

**Authors:** Aleksandra S. Orina, Olga P. Gavrilova, Nadezhda N. Gogina, Philipp B. Gannibal, Tatiana Yu. Gagkaeva

**Affiliations:** 1 Laboratory of Mycology and Phytopathology, All-Russian Institute of Plant Protection, 196608 St. Petersburg, Russia; olgavrilova1@yandex.ru (O.P.G.); fgannibal@vizr.spb.ru (P.B.G.); t.gagkaeva@yahoo.com (T.Y.G.); 2 Laboratory of Biochemical Analysis, All-Russian Scientific Research and Technological Institute of Poultry, 141311 Sergiev Posad, Russia; n.n.gogina@mail.ru

**Keywords:** grain, *Alternaria*, fungi, DNA, mycotoxins, co-occurrence

## Abstract

*Alternaria* fungi dominate the grain microbiota in many regions of the world; therefore, the detection of species that are able to produce mycotoxins has received much attention. A total of 178 grain samples of wheat, barley and oat obtained from the Urals and West Siberia regions of Russia in 2017–2019 were included in the study. Grain contamination with *Alternaria* fungi belonging to sections *Alternaria* and *Infectoriae* was analysed using qPCR with specific primers. The occurrence of four mycotoxins produced by *Alternaria*, AOH, AME, TEN, and TeA, was defined by HPLC-MS/MS. *Alternaria* DNA was found in all analysed grain samples. The prevalence of DNA of *Alternaria* sect. *Alternaria* fungi (range 53 × 10^−4^–21,731 × 10^−4^ pg/ng) over the DNA of *Alternaria* sect. *Infectoriae* (range 11 × 10^−4^‒4237 × 10^−4^ pg/ng) in the grain samples was revealed. Sixty-two percent of grain samples were contaminated by at least two *Alternaria* mycotoxins. The combination of TEN and TeA was found most often. Eight percent of grain samples were contaminated by all four mycotoxins, and only 3% of samples were free from the analysed secondary toxic metabolites. The amounts varied in a range of 2–53 µg/kg for AOH, 3–56 µg/kg for AME, 3–131 µg/kg for TEN and 9–15,000 µg/kg for TeA. To our knowledge, a new global maximum level of natural contamination of wheat grain with TeA was detected. A positive correlation between the amount of DNA from *Alternaria* sect. *Alternaria* and TeA was observed. The significant effects of cereal species and geographic origin of samples on the amounts of DNA and mycotoxins of *Alternaria* spp. in grain were revealed. Barley was the most heavily contaminated with fungi belonging to both sections. The content of AOH in oat grain was, on average, higher than that found in wheat and barley. The content of TEN in the grain of barley was lower than that in wheat and similar to that in oat. The content of TeA did not depend on the cereal crop. The effect of weather conditions (summer temperature and rainfall) on the final fungal and mycotoxin contamination of grain was discussed. The frequent co-occurrence of different *Alternaria* fungi and their mycotoxins in grain indicates the need for further studies investigating this issue.

## 1. Introduction

*Alternaria* Nees is a genus of ubiquitous fungi appearing on a wide range of substrates [1]. Most *Alternaria* species are pathogens responsible for plant diseases [2], but others can also live in plant tissue asymptomatically as endophytes [3,4]. Infection of crops with *Alternaria* spp. is common and can cause significant economic losses [5]. The contamination of crops with *Alternaria* fungi and the subsequent accumulation of their toxic metabolites in food and feed have been thoroughly investigated [6,7].

Many recent studies have demonstrated the predominance of *Alternaria* spp. in the mycobiota of cereal grains grown around the world [8,9,10,11,12,13]. Moreover, most of these *Alternaria* strains belong to *A.* section *Alternaria* Lawrence, Gannibal, Peever & Pryor and *A.* sect. *Infectoriae* Woudenb. & Crous [10,14], whereas species from sections *Pseudoalternaria* D.P. Lawr., Rotondo & Gannibal and *Panax* D.P. Lawr., Gannibal, Peever & B.M. Pryor appear sporadically [15,16,17].

In addition to the classic mycological methods for determining grain contamination with fungi, molecular approaches based on polymerase chain reaction (PCR), including real-time PCR, are currently actively used. The method allows quick and objective assessment of the quantitative presence of various fungal species in grain based on the content of their DNA, thereby eliminating errors at the taxonomic level [8,18,19].

*Alternaria* mycotoxins are widely found in a variety of food and feed [20]. The number of mycotoxins produced by *Alternaria* fungi has reached at least 70 compounds [21]. The most common of them in grain are alternariol (AOH), alternariol monomethyl ether (AME), tentoxin (TEN), and tenuazonic acid (TeA), which are often analysed and detected [14,22,23,24,25,26]. Presumably, AOH and AME have genotoxic, mutagenic and carcinogenic effects in humans and animals [20]. TeA is an inhibitor of protein synthesis and is a more toxic compound than AOH and AME [27]. TEN is relatively weakly toxic to mammals, but it is also a nonspecific phytotoxin and inhibits the development of chloroplasts, causing chlorosis of host plant tissues [27].

As a result of toxicological studies, it was proposed to introduce a restriction on the content of TeA at 500 µg/kg in infant food [28]. In June 2019, a draft EU Commission Recommendation on the monitoring of three *Alternaria* mycotoxins (AOH, AME, TeA) in food was issued: benchmark values in cereal-based foods for infants and children were 5 µg/kg for AOH and AME and 500 µg/kg for TeA [29]. There is no state standard regulating the content of *Alternaria* mycotoxins in food and feed in Russia.

The monitoring of grain infection with *Alternaria* fungi is always relevant because these organisms are able to produce mycotoxins, which can negatively affect grain consumers [27,30]. The potential for the production of mycotoxins by *Alternaria* spp. strains may differ significantly and depends greatly on environmental conditions [30,31]. The *Alternaria* species belonging to various *Alternaria* sections differ significantly in their toxin-producing ability [30,32,33,34] and pose different risks when contaminating food.

In Russia, several studies of occurrence of *Alternaria* fungi and their mycotoxins in grain cultivated in different regions have been carried out [10,14,35,36]. Previously, mycotoxins produced by *Alternaria* fungi were detected in grain samples mostly obtained from the central and southern regions of the European part of Russia and from the Urals; at the same time, information on grain contamination with *Alternaria* mycotoxins in Siberia was fragmentary [14,35,36]. It was noted that grain samples from the main grain-producing regions of Russia, such as the Southern European and North Caucasus regions, are less contaminated with *Alternaria* mycotoxins than those from the Central European, Volga, Urals and Siberian regions [36].

The main grain crop cultivated in the Urals and West Siberia regions is spring wheat, which is grown on ~60% of the total sown area of cereal crops in Russia. Oats (~50%) and barley (~24%) are also widely grown in these regions [37].

The aims of the present study were to determine the contamination of grain of spring wheat, barley and oat grown in the Urals and West Siberia regions of Russia in 2017–2019 by *Alternaria* fungi and their mycotoxins and to reveal the key factors affecting their distribution.

## 2. Results

### 2.1. The Approbation of The qPCR Protocol

The parameters of the adapted qPCR for analysis of the DNA content of *A.* sect. *Alternaria* fungi were 99.0% efficient, R^2^ = 0.999, and a slope of −3.35. The parameters of the adapted qPCR for analysis of the DNA content of *A.* sect. *Infectoriae* were 101.1%, R^2^ = 0.997, and slope −3.30. The level of specificity of the analysis is presented in Table 1. The amplification of the target DNA was detected at 11.2 ± 0.6 cycles for primers AAF2/AAR3 and at 21.1 ± 0.7 cycles for primers AinfF3/AinfR4. Nonspecific DNA amplification was detected after 32 cycles for both primer sets.

### 2.2. Content of Alternaria DNA in the Grain Samples

The qPCR results showed that all analysed grain samples were contaminated with DNA from both *Alternaria* sections. The amounts of *A.* sect. *Alternaria* DNA varied from 53 × 10^−4^ to 21,731 × 10^−4^ pg/ng, while the amounts of *A.* sect. *Infectoriae* DNA were in the range of 11 × 10^−4^–4237 × 10^−4^ pg/ng (Table 2).

Obviously, the amounts of DNA from *Alternaria* fungi belonging to sect. *Alternaria* were significantly more abundant in all analysed grain samples than in *A.* sect. *Infectoriae*. The differences in the average amounts reached 2.2–16.2 times. At the same time, a positive correlation (r = 0.46, *p* < 0.001) was found between the amounts of *Alternaria* fungi DNA from these two sections.

### 2.3. Content of Alternaria Mycotoxins in the Grain Samples

Five grain samples (four barley and one wheat) were free of *Alternaria* mycotoxins. Most grain samples (62%) were contaminated with a combination of any two mycotoxins. However, 16% and 8% of grain samples were contaminated by three (mostly the combination of AOH + TEN + TeA) and four mycotoxins, respectively.

Generally, mycotoxin AOH was found in 27% of grain samples, AME was detected in 12% of grain samples, and their content ranged from 2‒53 and 3‒56 µg/kg, respectively (Table 3). TEN turned out to be the most common *Alternaria* mycotoxin and was found in 90% of grain samples at levels of 3‒131 µg/kg. TeA was found in 85% of grain samples, and its content was 9‒14,963 µg/kg, which on average exceeded the content of other analysed *Alternaria* mycotoxins by 10‒27 times. The proportion of grain samples containing TeA in amounts >500 µg/kg, which is higher than the future recommended limit [28], was 4% of all analysed samples. Most of them originated from West Siberia. The maximum contents of *Alternaria* mycotoxins were detected in the grain samples from West Siberia: AOH—oat, Novosibirsk oblast, 2019; AME—wheat, Krasnoyarsky Krai, 2017; TEN—wheat, Altai Krai, 2017; and TeA—wheat, Altai Krai, 2018.

The combination of TEN and TeA was identified most often. The co-occurrence of AOH and AME was detected fairly rarely, and, as a rule, the AME content was lower than the AOH content. A significant positive correlation (r = 0.66, *p* < 0.001) between the contents of these two mycotoxins was revealed. As expected, a slight positive correlation between the contents of AOH and TeA was also detected (r = 0.17, *p* < 0.02).

A strong positive correlation between the content of *A.* sect. *Alternaria* DNA and TeA was revealed (r = 0.63, *p* < 0.001), which allows us to assume that these fungi are the main producers of this mycotoxin in cereal grains in the observed territory. The connection between the amount of *A.* sect. *Infectoriae* DNA and TEN contents was revealed (r = +0.31, *p* = 0.008) when the grain samples from the Urals were analysed separately. No other significant correlations between the content of mycotoxins and their potential producers were established.

### 2.4. Factors Affecting Grain Contamination

The results of the statistical analysis revealing the impact of different factors on the final content of fungal DNA in grain samples are summarized in Table 4.

The key factors affecting the contamination of grain with *A.* sect. *Alternaria* fungi were the cereal species and geographic origin of the samples. At the same time, the incidence of *A.* sect. *Infectoriae* fungi in grain samples was significantly related to cereal species and weather conditions prevailing in crop years. In the case of mycotoxin contamination of grain, the origin of the samples was not significant, in contrast to the other two factors. The cereal species confidently affected grain contamination with AOH and TEN, and the crop year conditions were important for the accumulation of AME and TEN.

#### 2.4.1. The Effect of Cereal Crop Species

**Figure 1:**
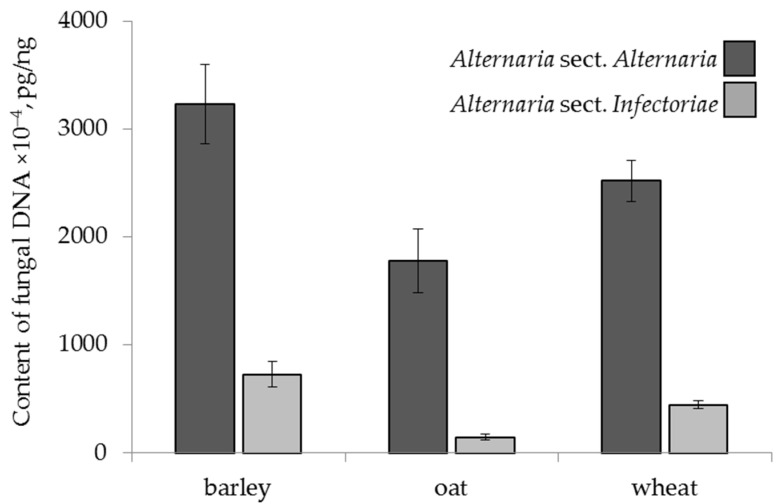
The DNA content of two sections of *Alternaria* fungi in the grain samples of the different cereals. The bars indicate confidence intervals with a 95% significance level.

The amounts of *Alternaria* spp. DNA belonging to the two sections differed significantly by cereal grain (Figure 1). The barley contained significantly more *A.* sect. *Alternaria* DNA (3233 × 10^−4^ pg/ng) than oat (1781 × 10^−4^ pg/ng) and wheat (2521 × 10^−4^ pg/ng). The highest average amount of *A.* sect. *Infectoriae* DNA was also found in barley (729 × 10^−4^ pg/ng), which was 1.6 times more than that in wheat (445 × 10^−4^ pg/ng) and 4.9 times more than that in oat (148 × 10^−4^ pg/ng).

The comparison of different cereals according to their contamination with *Alternaria* mycotoxins revealed that the occurrence of all mycotoxins was more frequent in oat grain (Table 5).

**Figure 2:**
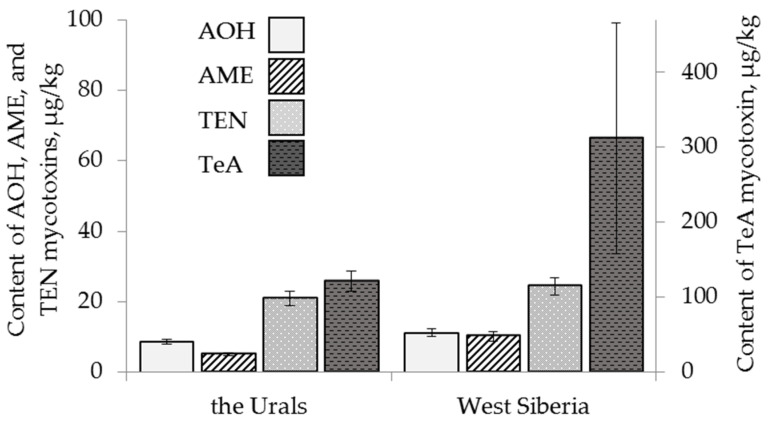
The content of *Alternaria* mycotoxins in the grain samples of the different cereals. The bars indicate confidence intervals with a 95% significance level. AOH: alternariol, AME: alternariol monomethyl ether, TEN: tentoxin, TeA: tenuazonic acid.

The content of AOH in oat grain samples was significantly higher (18 ± 5 µg/kg) than that found in wheat and barley. Barley was less contaminated with AOH and TEN than wheat (Figure 2). The mycotoxin AME was present only in one barley sample. The content of TEN in barley samples was, on average, lower (15 ± 2 µg/kg) than that in wheat and similar to that in oat. The difference between the average amounts of AME and TEN in wheat and oat grain samples was not detected. The content of TeA did not differ on average in the grain samples of all three cereals and reached 261 ± 133 µg/kg for wheat, 120 ± 20 µg/kg for barley and 309 ± 112 µg/kg for oat.

#### 2.4.2. The Effect of Geographical Origin

**Figure 3:**
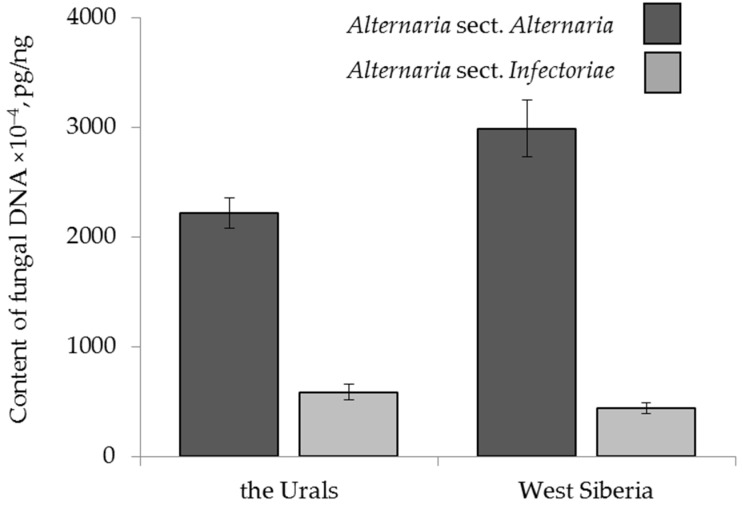
The DNA content of two sections of *Alternaria* fungi in the grain samples collected from the Urals and West Siberia in 2017–2019. The bars indicate confidence intervals with a 95% significance level.

The amount of *A.* sect. *Alternaria* DNA in grain from the Urals averaged (2215 ± 139) × 10^−4^ pg/ng, while in grain from West Siberia, the content of these fungi was significantly higher at (2989 ± 262) × 10^−4^ pg/ng. The amounts of *A.* sect. *Infectoriae* DNA in grain samples from the Urals and West Siberia were lower, and there was no significant difference between them: (587 ± 139) × 10^−4^ and (440 ± 90) × 10^−4^ pg/ng, respectively (Figure 3).

Mycotoxins AOH and AME were more frequent in grain from the West Siberia region (30% and 15% of samples, respectively) than in grain from the Urals (23% and 9%, respectively). The frequency of occurrence of TEN and TeA in grain from two regions was similar: TEN in 91% of the samples from the Urals and in 89% of the samples from West Siberia and TeA in 85% of the samples in each region. The contents of AOH, AME and TEN in grain from West Siberia (11, 10 and 21 µg/kg), on average, were similar to those in grain from the Urals (9, 5 and 24 µg/kg). At the same time, the TeA content in grain from West Siberia was 312 ± 154 µg/kg, which was 2.6 times higher than that in grain from the Urals (121 ± 14 µg/kg), but this difference was not significant.

#### 2.4.3. The Effect of Year

The years 2017–2019 were characterized by different weather conditions: a gradual decrease in the average summer temperature by 0.5 °C was noted during this period. In 2018, there was 25‒35% less precipitation than in 2017 and 2019.

**Figure 4:**
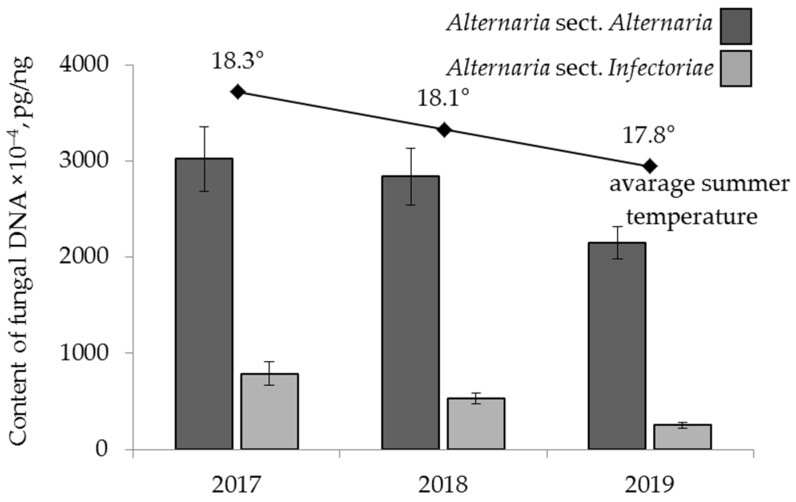
The DNA content of two sections of *Alternaria* fungi in the grain samples. The bars indicate confidence intervals with a 95% significance level.

The amounts of DNA from the analysed fungi in the grain harvested in these years varied significantly. A substantial decrease in the amount of *A.* sect. *Infectoriae* DNA in grain, from 789 × 10^−4^ pg/ng in 2017 to 252 × 10^−4^ pg/ng in 2019, was observed (Figure 4). A similar trend was also found for the amount of *A.* sect. *Alternaria* DNA, from 3021 × 10^−4^ pg/ng in 2017 to 2150 × 10^−4^ pg/ng in 2019.

The analysis of weather conditions during the vegetation period of the examined cereals in the three years investigated here revealed that only the average rainfall in July had a significant effect on the contamination of grain with fungi of *A.* sect. *Infectoriae* (r = 0.24, *p* < 0.001). At the same time, the DNA content of *A.* sect. *Alternaria* was positively correlated with average temperature (r = 0.31, *p* < 0.001) and rainfall (r = 0.19, *p* < 0.001) in June. In July, the correlations were negative, and the relationships between the average monthly temperature and rainfall and the DNA content of *A.* sect. *Alternaria* obtained in grain were r = −0.19 at *p* = 0.01 and r = −0.21 at *p* = 0.01. Rainfall in August also negatively affected grain infection by the fungi (r = −0.17, *p* = 0.005).

**Figure 5:**
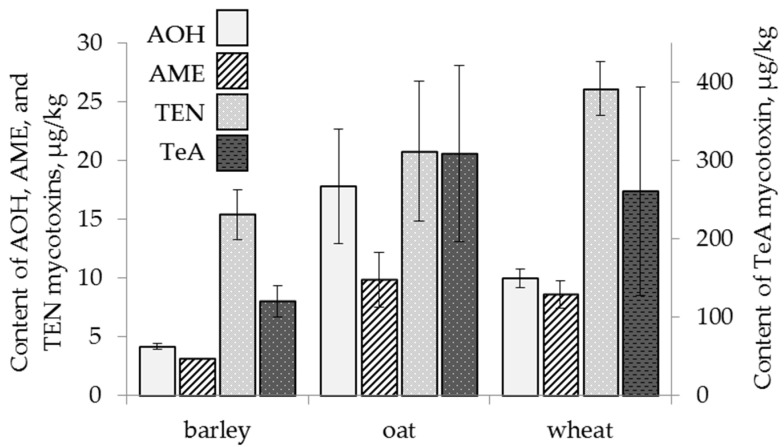
The content of *Alternaria* mycotoxins in the grain samples. The bars indicate confidence intervals with a 95% significance level. AOH: alternariol, AME: alternariol monomethyl ether, TEN: tentoxin, TeA: tenuazonic acid.

Among the analysed mycotoxins, the content of TEN showed a similar trend, decreasing both in occurrence in grain (from 100% to 80% of samples) and in the average content (from 36 µg/kg to 9 µg/kg) in the period 2017–2019 (Figure 5). The content of TEN had a significant correlation only with the average temperature in June (r = +0.25, *p* < 0.001) and did not depend on climatic factors in other months. The significant correlation between the content of other mycotoxins in the grain and weather conditions during the vegetation period of 2017–2019 was not established.

The lowest occurrence of AOH and AME in the grain was observed among samples harvested in 2018 (22% and 9% of samples, respectively), while the highest occurrence of AOH and AME in the grain was found in samples from 2017 (39% and 22% of samples). Among grain samples harvested in 2019, AOH and AME were detected in 25% and 11% of the samples, respectively. The average AME content in the grain harvested in 2018 was 4 µg/kg, while in 2019 and 2017, this value was 2.0–2.9 times higher.

The occurrence of TeA in the samples harvested in 2017 and 2019 turned out to be similar (95% and 98% of the samples), while in the samples harvested in 2018, it was lower (73%). However, in the samples harvested in 2018, the average TeA content (409 µg/kg) was 3.1–3.8 times higher than that in the samples harvested in 2017 and 2019.

## 3. Discussion

*Alternaria* spp. are abundantly present in the grain mycobiota in the Urals and West Siberia regions of Russia [14,38] and neighbouring territories such as Kazakhstan [39] and China [34]. Until recently, *A.* sect. *Alternaria* fungi were thought to be evenly distributed in Russia, while *A.* sect. *Infectoriae* species were often found in Europe, rarely in the Urals and Siberia, and were absent in the Russian Far East [40]. However, our study demonstrated the significant abundance of *A.* sect. *Infectoriae* fungi in grain in the Urals and West Siberia regions, which is consistent with the recent results of a mycological analysis of grain grown in these regions [34].

The accurate morphological identification of *Alternaria* spp. is difficult due to the vagueness of species boundaries [32,41,42]. In this research, for the first time, the use of specific primers for the molecular detection of fungi belonging to two *Alternaria* sections, *Alternaria* and *Infectoriae*, allowed us to establish the contamination of grain with those fungi through DNA content and to comprehensively analyse the occurrence of different *Alternaria* fungi in cereals. The specificity of primers previously developed for qualitative analysis [43,44] was confirmed during qPCR. Thus, the quantitative detection of *Alternaria* DNA in grain was carried out at the level of two sections, *Alternaria* and *Infectoriae*, whereas earlier, a similar study was focused on the genus level [8]. The difference in Ct of detection is explained by the location of the annealing sites in the genome. The primer set AAF2/AAR3 amplified the fragment of the high-copy ITS region [43]. The primer set AinfF3/AinfR4 was designed based on the sequence of specific DNA fragments obtained by UP-PCR, which is probably a single- or oligo-copy genome locus [44].

DNA of *Alternaria* fungi of both sections was found in all analysed grain samples. A significant positive correlation between the amounts of *A.* sect. *Alternaria* and *A.* sect. *Infectoriae* DNA in grain was revealed, which indicates that both groups of species need very similar conditions for their development. The predominance of *A.* sect. *Alternaria* fungi over *A.* sect. *Infectoriae* in the analysed grain samples was observed: the amount of *Alternaria* spp. DNA from sect. *Alternaria* was more than 5 times higher than that of *A.* sect. *Infectoriae*. Notably, *A.* sect. *Infectoriae* fungi are often characterized by a higher growth rate [45], and these fungi are dominant in wheat, barley and oats grown in Norway [46] and Australia [9]. At the same time, *A.* sect. *Alternaria* fungi form a denser pigmented mycelium [31] and abundant sporulation [47], which likely provides a competitive advantage in the conditions of the Urals and West Siberia. The prevalence of *A.* sect. *Alternaria* fungi was also noted in grain from Hebei Province in China [34]. These fungi are widespread in wheat and barley grains in Asia and produce the main *Alternaria* mycotoxins, such as AOH, AME, and TeA [34,48]. In our study, a strong positive correlation between the contents of *A.* sect. *Alternaria* DNA and TeA was revealed, which indicates the key role of these fungi as the main producers of TeA in grain, at least in the observed territory. However, there was no reliable connection between the amount of *A.* sect. *Infectoriae* DNA and any analysed mycotoxins in grain. The proportion of *Alternaria* spp. from the sections *Alternaria* and *Infectoriae* in the grain mycobiota can vary and largely depends on weather conditions, as well as on the host plant [10,46], which finally determines the contamination of grain with mycotoxins.

Most grain samples were contaminated with at least two *Alternaria* mycotoxins, and only 3% of analysed grain samples were free from mycotoxins. Recently, the predominance of *Alternaria* mycotoxins (mainly TEN) in grain samples from the Urals and Siberia region of Russia has also been shown [14,36]. In our study, TeA and TEN were found more often than AOH and AME in the grain samples from both regions, which corresponds to previous reports indicating these *Alternaria* mycotoxins are the most common in grain grown in Asia [22,34].

Consistent with previously published data [23,49], the amounts of AOH, AME and TEN were, as a rule, lower than the amounts of TeA in the same grain samples and did not exceed 100 µg/kg. The amounts of AOH and AME in the analysed grain reached 53 µg/kg and 56 µg/kg, respectively, when, previously, the maximum detected amount of AOH in Russia was 675 µg/kg in wheat from the North Caucasus region and 397 µg/kg in barley from the Central European region [35]. The authors noted the unequal distribution of abundances of AOH in grain samples. Half of the data were less than the median value, and the largest amounts of AOH exceeded the threshold concentrations calculated for the 90th percentile, which indicated the possibility of its abnormally high accumulation.

Only in two grain samples did the amount of TEN exceed 100 µg/kg. TeA was detected in significant amounts, more than the recommended benchmark value of 500 µg/kg [29], in eight grain samples. Meanwhile, TeA is an acutely toxic substance with oral LD50 values ranging from 81 to 225 mg/kg bw for mice [50]. One wheat sample from West Siberia (Altai Krai) contained TeA in an extremely high amount of 15,000 µg/kg. To our knowledge, this is the highest recorded natural contamination of wheat grain with this mycotoxin. Previously, the highest amounts of TeA were detected in wheat grain in Argentina—8814 µg/kg [51], Germany—4179 µg/kg [52], and China—6432 µg/kg [53], 3331 µg/kg [22] and 3634 µg/kg [34]. In our study, the grain sample of wheat from Altai Krai that was contaminated by the maximal amount of TeA also contained the maximum quantity of *A.* sect. *Alternaria* DNA.

The co-occurrence of AOH and AME with TeA was found in 10% of the analysed grain samples. This phenomenon was observed repeatedly, and it has been shown that the TeA content was higher than that of other mycotoxins [22,23,26,34,54]. A significant positive correlation between AOH and AME, which are derivatives of the same chemical precursor dibenzopyrone, and between AOH and TeA was revealed. Recently, similar statistically significant correlations between different *Alternaria* toxins were revealed in wheat grain samples and wheat-based products from China [22,55]. The main danger of the co-occurrence of *Alternaria* mycotoxins is synergistic for additive interactions between these toxic metabolites that may enhance the negative health effects of consumers [55].

In our study, the significant effects of cereal species, geographic origin of samples and weather conditions during the vegetation season on fungal and mycotoxin contamination were established.

According to quantitative analysis of average DNA levels, barley grain was the most heavily contaminated by *Alternaria* spp., as opposed to oats. The mycological analysis of grain grown in Greece showed a similar result: barley grain was the most heavily infected with *Alternaria* fungi, in contrast with wheat and oat grain [56].

Very likely, the amount of fungal DNA in grain barely reflects the amounts of corresponding mycotoxins. Over three years, oat grain samples were, on average, the most heavily contaminated with mycotoxins, despite being the least infected. Barley grain was the least contaminated cereal and contained the lowest average amounts of mycotoxins. A similar result was obtained when 76 grain samples from southern Norway were analysed: the occurrence of TEN and TeA in oat grain was significantly higher than that in barley and wheat, although the detected contents of these mycotoxins were low [54]. The analysis of 110 grain samples from Latvia revealed the more significant contamination of oat grain by AOH and AME compared to rye, winter wheat, and barley [57]. Under laboratory conditions during artificial inoculation, a significant effect of the grain substrate on the accumulated levels of mycotoxin by *A. alternata* (Fr.) Keissl. strains has also been shown [58].

The prevalence of AOH and its accumulated amounts, in comparison with TeA and AME, in barley grain was also revealed, but the authors noted a significant effect of weather conditions on the occurrence of mycotoxins [25].

The abundance of fungi belonging to *A.* sect. *Alternaria* was significantly higher in grain samples from West Siberia than in grain samples from the Urals. Perhaps this can be explained by the difference in climatic conditions: the summer temperature in West Siberia, over a three-year average, was 1.3 °C higher. The difference between the contamination of grain with fungi of *A.* sect. *Infectoriae* from the two observed regions was not revealed.

The analysis of weather conditions during different vegetation periods revealed that the DNA content of *A.* sect. *Alternaria* fungi in grain positively correlated with average temperature and average rainfall in June but had a negative relation in July‒August. Warm and humid weather during the periods of wheat flowering, sprouting and heading (late April to June) in Anhui Province in China was favourable to cereal infection by *Alternaria* fungi [22]. In the case of DNA analysis of *A.* sect. *Infectoriae*, only the abundance of the average rainfall in July had a significant effect on infection of grain. Among the four mycotoxins, a significant positive correlation with the average temperature in June was revealed only for TEN.

Weather influenced the level and distribution of *Alternaria* mycotoxin contamination of grain in Argentina when wetter conditions during the wheat‒growing season with heavy rainfall in August and December led to high concentrations of mycotoxins [51]. Regardless of climatic conditions, TeA was always present in wheat grain from Serbia, with different frequencies and at different concentrations [59], but in the year with the highest amount of precipitation (up to 170% increased amount of rainfall compared to long-term annual precipitation data), fungal growth and production of AOH and TeA increased and were observed to have the highest values.

Previously, it was revealed that the number of *Alternaria* spp. conidia in field air and the amount of *Alternaria* DNA in cereal plant tissue were higher with a lower humidity and a higher temperature [60]. Most likely, the penetration of *Alternaria* spp. into the grains occurs at the early stages of plant growth in June, which is favoured by high temperature and the absence of competitors. The revealed trends should be compared with the results obtained under other climatic conditions for other cereal crops.

## 4. Conclusions

For the first time, grain contamination with *Alternaria* fungi was analysed using qPCR at the section level. The ubiquitous presence of *Alternaria* spp. belonging to sections *Alternaria* and *Infectoriae* and their mycotoxins in the grain of wheat, barley and oats in the Urals and West Siberia was demonstrated. The prevalence of DNA of *A.* sect. *Alternaria* fungi over the DNA of *A.* sect. *Infectoriae* in the grain samples was revealed.

The 97% grain samples were contaminated with at least one *Alternaria* mycotoxin. The new global maximum level of natural contamination of wheat grain with TeA (15,000 µg/kg) was detected. The relationship between grain infection, which was determined as the abundance of fungal DNA, and the content of mycotoxins in certain species of cereal was established. Frequent co-contamination of grain with *Alternaria* mycotoxins and the revealed extremely high TeA content in wheat grain showed the necessity of further research addressing this problem as well as the development of measures to control grain contamination with *Alternaria* mycotoxins.

## 5. Materials and Methods

### 5.1. Grain Samples and Weather Conditions of Vegetation Seasons in the Analysed Regions

The sampling was carried out by the specialists of the Russian Agricultural Center in accordance with the standard protocol adopted in Russia. In total, 75 grain samples of cereal crops grown in four locations in the Urals (Chelyabinsk, Kurgan, Tyumen, and Sverdlovsk regions) and 103 grain samples from five locations in West Siberia (Altai Krai, the southern part of Krasnoyarsk Krai, Kemerovo, Novosibirsk, and Omsk regions) in 2017–2019 were analysed. Grain samples were represented by 116 wheat, 49 barley and 13 oat samples.

The available data on climatic conditions in the Urals and West Siberia regions in 2017–2019 are presented in Table 6.

### 5.2. Grinding Samples

From each grain sample 20 g was taken and then homogenized in a grinding chamber of a batch mill with a tube mill control (IKA, Königswinter, Germany) for extraction of DNA and mycotoxins. The ground cereal flour was stored at –20 °C until DNA and mycotoxins were extracted.

### 5.3. DNA Extraction and Concentration Measurement

The extraction of total DNA from 200 mg of flour sample, as well as from the mycelium of *Alternaria* spp. strains, was performed using the Genomic DNA Purification Kit (Thermo Fisher Scientific, Vilnius, Lithuania) according to the adapted protocol. Total concentrations of DNA were determined using a Qubit 2.0 Fluorometer with a Quant-iT dsDNA HS Assay Kit (Thermo Fisher Scientific, Waltham, MA, USA). The DNA of the fungal strains was diluted to a concentration of 10 ng/µL and used to construct a calibration curve. The DNA isolated from analysed grain samples was aligned to 2‒50 ng/µL.

### 5.4. Detection of Fungal DNA Content in Grain Using qPCR

The amounts of DNA from *Alternaria* fungi belonging to sections *Alternaria* and *Infectoriae* were determined using SYBR Green qPCR with primers designed for the qualitative detection of *Alternaria* spp. belonging to the two sections and adapted for the quantitative analysis of their presence in the grain samples (Table 7). The reaction was carried out in a 20 µL volume containing 4 µL of 5×qPCRmix-HS SYBR master mix (Evrogen, Moscow, Russia), each primer at 500 nM, and 2 µL of DNA solution. All qPCR assays were performed using a CFX 96 Real-Time System thermocycler (BioRad, Hercules, CA, USA). Fold differences and standard errors were calculated from the Ct values using the Bio-Rad CFX Manager 1.6 software package. To determine the sensitivity and specificity of the analysis, the DNA of 12 strains stored in the Laboratory of Mycology and Phytopathology of the All-Russian Institute of Plant Protection (St. Petersburg, Russia) were selected. Among the samples were strains of the genus *Alternaria* of the sections *Alternaria*, *Infectoriae*, and *Pseudoalternaria*, as well as fungi of the genera *Bipolaris* Shoemaker, *Cladosporium* Link, *Fusarium* Link, and *Trichothecium* Link, common representatives of the cereal grain mycobiota. The content of *Alternaria* spp. DNA in grain samples was presented as the ratio of fungal DNA to total DNA isolated from each sample (pg/ng). The quantification value of 5 × 10^−4^ pg of fungal DNA in one ng of total DNA was established as the threshold of the low limit of DNA in a sample, which can be quantitatively determined with high precision.

### 5.5. Analysis of Secondary Metabolites of Fungi by HPLC-MS/MS

The mycotoxins analysis was carried out according to the standard method [61]. Metabolites were extracted from 5 g of cereal flour by adding 20 mL of extraction solvent (acetonitrile/water/acetic acid, 79:20:1, *v*/*v*/*v*) and mixing on a PSU-20 rotary shaker (Biosan, Riga, Latvia) for 90 min. Two independent extractions were prepared for each sample. Then, the extracts were centrifuged for 2 min at 3000 rpm (Polycom CLn-16, Moscow, Russia). Five hundred microlitres of each extract without any purification was transferred into glass vials, and 500 μL of a solution of acetonitrile:water:acetic acid 20:79:1 was added. Then, the vials were sealed and shaken for 30 s on a Vortex Genius3 (IKA, Germany). For analysis, 5 μL of each extract solution was taken by an Agilent autosampler (Agilent Technologies, Germany). Two injections were performed in the LC-MS.

The elution was carried out in binary gradient mode with a flow rate of 1000 μL/min. Both mobile phases contained 5 mM ammonium acetate and were composed of methanol/water/acetic acid 10:89:1 (*v*/*v*/*v*; eluent A) and 97:2:1 (*v*/*v*/*v*; eluent B), respectively. After an initial time of 1.5 min at 100% A, the proportion of B was increased linearly to 50% within 3 min. Further linear increase of B to 100% within 12.0 min was followed by a hold time of 5 min at 100% B and 3.5 min column re-equilibration at 100% A. The injection volume was 5 μL. ESI-MS/MS was performed in the scheduled multiple reaction monitoring (sMRM) mode negative polarity in two separate chromatographic runs. The target cycle time was 1000 ms, the MS pause time was 3 ms, and the detection window width was 40 and 52 s in the negative ESI mode.

Detection and quantification of four mycotoxins produced by *Alternaria* fungi were performed on an AB SCIEX Triple Quad™ 5500 MS/MS system (Applied Biosystems, Foster City, CA, USA) equipped with a TurboV electrospray ionization (ESI) source and a 1290 series UHPLC system (Agilent Technologies, Waldbronn, Germany). Chromatographic separation was carried out at 25 °C using a Gemini^®^ C18 column, 150 × 4.6 mm (Phenomenex, Torrance, CA, USA). The contents of four mycotoxins, AOH, AME, TEN, and TeA, produced by *Alternaria* fungi were determined in the extracts. For the quantitative detection of mycotoxins in grain the matrix-matched calibration techniques using standard solutions of mycotoxins (Romer Labs, Tulln, Austria) were applied. The parameters of the HPLC-MS/MS method for the analysed mycotoxins are presented in Table 8. 

The limit of detection (LOD) for the analysed mycotoxins was established by 20 measurements of each pure matrix and calculation of the average value. The limit of quantification (LOQ) for the analysed mycotoxins was determined by adding each analysed mycotoxin to a pure matrix. When the S/N (signal to noise) value of 20 parallel measurements was above five and the reproducibility was above 80%, the LOQ for each matrix was established. The LOD and LOQ for the analysed mycotoxins are presented in Table 9.

### 5.6. Statistical Analysis

Microsoft Excel 2010 was used to calculate the average values and the confidence intervals at a significance level of *p* < 0.05. The analysis of data variance and the correlation analysis of quantitative traits expressed by the Pearson coefficient (r) at a significance level of *p* < 0.05 were carried out with the STATISTICA 10.0 program.

## Figures and Tables

**Table 1:** Specificity of qPCR for detection of *Alternaria* spp. DNA.

Strain ID	Fungi	Results of qPCR with Primer Pairs (Ct ^1^)	
		AAF2/AAR3	AinfF3/AinfR4
MFP556081	*A. tenuissima* (sect. *Alternaria*)	10.9	33.7
MFP028011	*Alternaria* sp. (sect. *Alternaria*)	11.5	34.4
MFP094121	*Alternaria* sp. (sect. *Pseudoalternaria*)	34.6	35.5
MFP457051	*Alternaria* sp. (sect. *Pseudoalternaria*)	32.9	37.7
MFP094331	*Alternaria* sp. (sect. *Infectoriae*)	34.1	20.8
MFP778011	*Alternaria* sp. (sect. *Infectoriae*)	35.5	21.5
MFG59013	*Bipolaris sorokiniana*	n.d. ^2^	n.d.
MFG232100	*Cladosporium* sp.	n.d.	34.5
MFG60204	*Fusarium avenaceum*	n.d.	35.4
MFG102100	*F. culmorum*	n.d.	n.d.
MFG11039	*F. sporotrichioides*	n.d.	n.d.
MFG14000	*Trichothecium roseum*	n.d.	38.8

^1^ Ct—amplification cycle corresponding to the cross of fluorescence curve with the threshold; ^2^ n.d.—absence of amplification products.

**Table 2:** The content of *Alternaria* DNA in the grain samples of different cereals from the Urals and West Siberia.

Cereal	Region	Crop Year (Number of Samples)	The Range of *Alternaria* DNA Amounts ×10^−4^, pg/ng of Total DNA	
			*A.* sect. *Alternaria*	*A.* sect. *Infectoriae*
wheat	the Urals	2017 (13)	881–3079	117–2388
		2018 (23)	736–4580	36–1462
		2019 (10)	1165–3826	55–544
	West Siberia	2017 (26)	1027–6442	158–877
		2018 (21)	1213–21,731	91–1187
		2019 (23)	426–3851	35–1204
barley	the Urals	2018 (15)	497–5568	26–3616
		2019 (8)	361–7146	43–1051
	West Siberia	2017 (2)	12,721; 9695	4237; 2746
		2018 (15)	53–10,526	252–1472
		2019 (9)	820–4776	103–535
oat	the Urals	2018 (2)	1093; 1452	88; 326
		2019 (4)	313–3323	11–227
	West Siberia	2018 (5)	905–3472	79–327
		2019 (2)	624; 526	139; 91

**Table 3:** The content of mycotoxins produced by *Alternaria* fungi in the grain samples of different cereals from the Urals and West Siberia regions of Russia.

Region	Year of Crop (No. of Samples)	Number of Contaminated Samples/ Range of Mycotoxin Amounts, µg/kg			
		AOH	AME	TEN	TeA
wheat					
the Urals	2017 (13)	3/2–4	nd/0	13/4–48	13/15–226
	2018 (23)	6/3–26	3/5–5	21/3–79	16/35–454
	2019 (10)	1/15	nd ^1^/0	7/5–19	10/19–97
West Siberia	2017 (26)	13/2–44	9/3–56	26/9–131	26/17–545
	2018 (21)	7/3–14	2/3; 4	20/4–83	16/37–14,963
	2019 (23)	6/7–17	3/4–6	17/3–36	23/16–241
barley					
the Urals	2018 (15)	3/2–8	1/3	14/5–80	13/15–593
	2019 (8)	1/3	nd/0	7/5–10	7/14–570
West Siberia	2017 (2)	nd/0	nd/0	2/9; 12	nd/0
	2018 (15)	nd/0	nd/0	13/5–38	7/30–349
	2019 (9)	2/4; 5	nd/0	7/3–6	9/9–113
oat					
the Urals	2018 (2)	1/19	1/3	2/16; 21	2/65; 228
	2019 (4)	2/11; 13	2/4; 11	4/3–15	3/59–276
West Siberia	2018 (5)	1/4	nd/0	5/13–88	5/164–405
	2019 (2)	2/7; 53	1/22	2/15; 15	2/280; 1579

^1^ nd—none of the samples contained mycotoxin.

**Table 4:** The effect of different factors on the content of *Alternaria* DNA and mycotoxins in grain.

Factors	Analysed Parameters					
	*A.* sect. *Alternaria* DNA	*A.* sect. *Infectoriae* DNA	AOH	AME	TEN	TeA
cereal species	F ^1^ = 3.50 *p* = 0.03	F = 7.48 *p* = 0.001	F = 7.25 *p* = 0.0009	ns ^2^	F = 3.32 *p* = 0.04	ns
geographic origin	F = 6.03 *p* = 0.02	ns	ns	ns	ns	ns
crop year	ns	F = 12.03 *p* = 0.00001	ns	F = 3.06 *p* = 0.049	F = 9.48 *p* = 0.002	ns

^1^ F—value of Fisher test; *p*—significance level; ^2^ ns—marked not significant values.

**Table 5:** The occurrence of *Alternaria* mycotoxins in the grain samples of the different cereals.

Cereals (No. of Samples)	The Proportion of Samples Containing Mycotoxin, %			
	AOH	AME	TEN	TeA
Barley (49)	12	2	88	73
Oat (13)	46	31	100	92
Wheat (116)	31	15	90	90

**Table 6:** Weather data during the growing season of 2017–2019 in the Urals and West Siberia regions (https://rp5.ru/, accessed on 20 July 2021).

Region	Year	Month	Average Month Temperature, °C	Average Summer Temperature, °C	Rainfall, mm	Days with Precipitation
the Urals	2017	June	+16.6	+17.7	72	19
		July	+18.5		109	19
		August	+18.0		57	13
	2018	June	+15.0	+17.5	36	19
		July	+21.0		90	13
		August	+16.5		65	19
	2019	June	+15.9	+17.5	64	18
		July	+20.3		75	14
		August	+16.4		85	18
West Siberia	2017	June	+20.2	+18.9	50	14
		July	+19.0		94	23
		August	+17.5		69	16
	2018	June	+19.9	+18.6	64	16
		July	+18.9		52	14
		August	+17.1		26	13
	2019	June	+16.6	+18.1	48	16
		July	+19.3		83	12
		August	+18.5		61	17

**Table 7:** The primers and the protocols of quantitative PCR used in this study.

Target	The Primers	Primer Sequence (5′ → 3′)	Protocol	References
*A.* sect. *Alternaria*	AAF2	TGCAATCAGCGTCAGTAACAAA	50° for 2 min; 95° for 10 min; [95° for 15 s; 67° for 60 s; 72° for 5 s] × 40	[43]
	AAR3	ATGGATGCTAGACCTTTGCTGAT		
*A.* sect. *Infectoriae*	AinfF3	CTCGATGTCCGCCTCAGTAG	50° for 2 min; 95° for 10 min; [95° for 15 s; 65° for 60 s; 72° for 3 s] × 40	[44]

**Table 8:** Parameters of HPLC-MS/MS method.

Analyte	Retention Time, min	MS/MS Parameters				
		*m*/*z* Q1	*m*/*z* Q3 ^1^	DP (V) ^2^	CE (V) ^3^	CXP (V) ^4^
Alternariol (AOH)	9.67	257.0	213.0/215.0	−100	−34/−36	−11/−11
Alternariolmethylether (AME)	11.40	271.0	256.0/227.0	−95	−32/−50	−13/−9
Tentoxin (TEN)	8.84	413.3	141.0/271.1	−105	−30/−24	−11/−24
Tenuazonic acid (TeA)	8.04	196.1	139.0/112.1	−120	−28/−28	−7/−7

^1^ quantifier/qualifier specific product ions; ^2^ declustring potential; ^3^ collision energy; ^4^ cell exit potential.

**Table 9:** Precision, limits of detection (LODs), and limits of quantification (LOQ) for the analysed mycotoxins in cereal matrix.

Analyte	Precision (±), %	LOD, µg/kg	LOQ, µg/kg
Alternariol (AOH)	10–21		
wheat		0.79	2.39
barley		0.82	2.46
oat		0.82	2.60
Alternariolmethylether (AME)	7–23		
wheat		0.69	2.15
barley		0.70	2.15
oat		0.82	2.67
Tentoxin (TEN)	8–11		
wheat		0.79	2.00
barley		0.82	2.15
oat		0.82	2.22
Tenuazonic acid (TeA)	13–21		
wheat		6.3	14.80
barley		3.40	9.00
oat		18.44	51.25

## Data Availability

Data is contained within the article.
